# The cost of lost productivity due to premature lung cancer-related mortality: results from Spain over a 10-year period

**DOI:** 10.1186/s12885-019-6243-7

**Published:** 2019-10-23

**Authors:** Josep Darbà, Alicia Marsà

**Affiliations:** 1 Department of Economics, Universitat de Barcelona, Diagonal 696, 08034 Barcelona, Spain; 2 BCN Health Economics & Outcomes Research S.L., Travessera de Gràcia, 62, 08006 Barcelona, Spain

**Keywords:** Lung cancer, Years of potential productive life lost, Productivity costs, Human capital approach

## Abstract

**Background:**

Cancer mortality is one of the major causes of productivity loss; and within all cancer sites, malignant neoplasms of the lung continue to be the principal cancer-related cause of death in Spain, with a survival rate of only 10.7%. Thus its effects in labour productivity are a major concern and represent a great social impact. The objective of this study was to evaluate the productivity losses that occur as a result of premature deaths due to lung cancer in Spain.

**Methods:**

The human capital approach was used to calculate the costs derived from the premature mortality due to lung cancer, via the extraction of data on mortality, reference salaries and unemployment rates.

**Results:**

Deaths due to lung cancer represented the 28.90% and the 10.83% of all cancer-related deaths in 2017 in males and females respectively, with an increasing tendency in this last group. In addition, the YPPLL count increased in the study period among females. Lung cancer was responsible annually for 60,846 YPPLL, and productivity losses summed €13.1 billion over the 10 year period.

**Conclusions:**

The assessment of productivity losses due to lung cancer provides new information that may assist decision makers in the allocation of resources, reducing the burden it supposes in working-age individuals.

## Background

Lung cancer is the fourth most prevalent cancer among males in Spain, the ninth in women, and the leading cancer-related cause of death in males [1]. Worldwide, tracheal, bronchus and lung cancers were the leading cancer-related cause of death for both males and females in 2013 [2], indeed, in Spain, the 5 year survival rate is only 10.7% [3].

Lung cancer mortality peaks in individuals aged 75 to 79 years in developed countries as the United Kingdom [4]; however, worldwide, the highest rates are observed in males between 60 and 75 years of age [5]. Altogether, deaths prior 65 years of age have a notorious impact on economic productivity, in which cancer plays a great role [6]. In males, cancers of the trachea, bronchus and lung together were the leading cause of disability-adjusted life-years (DALYs), which combine health loss with premature mortality, accounting for 34.7 million DALYs in 2013, 62% of those in developing countries and 38% in developed countries [2].

Estimations of productivity loss provide valuable data for informed resource allocation. Distinct approaches are used to estimate lost productivity. The human capital approach is the majority method, based on the assumption that individuals have a potential productivity to their retirement age that is reduced due to illness or death, leading to losses that can be quantified [7]. Secondarily, the friction cost approach is centred on the losses that take place in the time it takes to replace a worker; this method aims to provide a more realistic calculation, however, it requires the use of a standard measurement of replacement time [8]. Finally, other methods exist focusing on different variables, for instance the willingness to pay approach values immaterial costs as pain and distress [9].

Altogether, predictions of cancer mortality-related productivity loss have been projected for various countries [10, 11]. In Spain, the estimated losses due to cancer temporary disability were €248.6 million in 2005 [12], added to the €2.5 billion in losses due to premature mortality estimated in 2009 [13]. The scale of such costs demonstrates the extent of the burden of cancer and the possible economic gains that could be reached by the implementation of informed policies that reduce the incidence of the cancers causing the most expenses.

The present study aimed to evaluate the losses in terms of productivity that occur as a result of premature deaths due to lung cancer in Spain.

## Materials and methods

### Study design

This study was developed based on the human capital approach to calculate the costs derived from premature mortality due to lung cancer, considering the income and contribution to the nation productivity of an individual that are prevented when a premature death occurs. This method was selected taking into account previous studies in the country, and looking to provide a calculation from the worker’s perspective.

Mortality data and reference salaries per age group were obtained from the Spanish National Statistics Institute (INE) [14, 15]. The years of potential productive life lost (YPPLLs) due to premature mortality from lung cancer were estimated by multiplying the number of lung cancer-specific deaths for a given age group by the expected productive years remaining for each group. Retirement age was fixed at 65 years.

To obtain an estimation of costs of premature mortality, age- and sex-specific annual wages from death age to age of retirement were used. YPPLL was corrected per age- and gender-specific unemployment rates [16] and an annual discount rate of 3% was applied to future income values. A sensitivity analysis was conducted considering two alternative discount rates (0 and 6%).

## Results

In total, 212,632 people died of lung cancer in Spain between 2008 and 2017, 69,225 during working age. Deaths due to lung cancer represented 28.90% of all cancer-related deaths in 2017 in males and 10.83% in females, with an increasing tendency in this last case (Table 1). The year 2008 displayed the highest number of YPPLLs for males, 49,654, while the highest number in females was observed the last year of the study period, 23,035 measured in 2016. Altogether, the average annual YPPLL count was 60,846.

**Table 1:** Indicators of deaths, portion of cancer-related deaths attributable to lung cancer and years of potential productive life lost (YPPLL) due to lung cancer

Year	2008	2009	2010	2011	2012	2013	2014	2015	2016	2017
Number of deaths										
males	17,150	17,279	17,285	17,479	17,661	17,559	17,194	17,239	17,598	17,241
females	3049	3122	3447	3579	3826	4105	4057	4357	4557	4848
Deaths at working age										
males	5613	5564	5520	5468	5535	5252	5064	5015	5004	4697
females	1290	1321	1495	1497	1657	1736	1680	1902	1918	1997
% of lung cancer related deaths										
males	28.43	26.61	27.94	33.61	26.95	27.59	34.38	25.46	25.65	28.90
females	7.68	8.29	8.39	10.54	9.37	9.75	10.22	9.98	10.91	10.83
YPPLL										
males	49,654	47,097	46,225	46,239	46,455	43,281	40,852	40,065	38,757	36,246
females	15,210	15,368	16,430	16,126	17,491	17,708	16,390	18,076	17,754	23,035

**Fig. 1:**
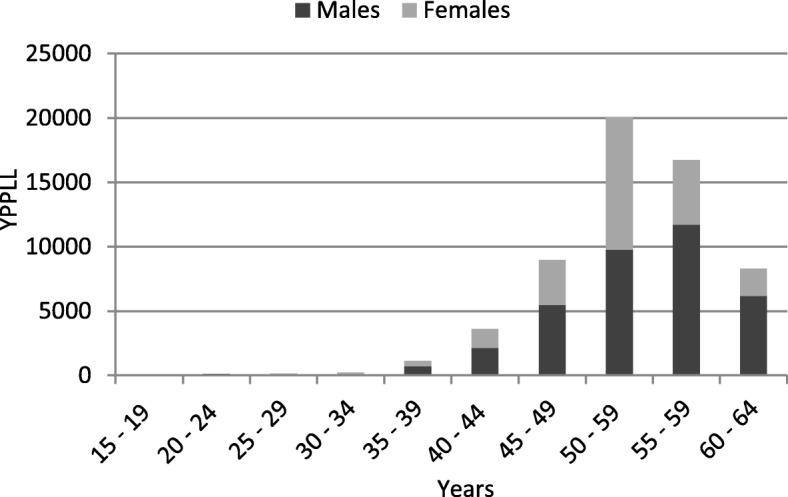
Years of potential productive life lost (YPPLL) due to premature mortality from lung cancer per age groups in 2017

The year 2017 was considered the reference year in the analysis of the age distribution of YPPLL. Premature mortality in the age period between 50 and 59 years accounted for the highest values of YPPLL (Fig. 1).

The costs of premature mortality were estimated, projecting productivity losses to retirement years and adjusting all calculations per age- and sex-specific annual wages. The analysis provided three estimations, a baseline, and the results of the sensitivity analysis. The accumulated productivity losses from 2008 to 2017 due to lung cancer were €13.1 billion (Table 2). The sensitivity analysis determined a range between €12.8 and €13.5 billion. The amount of losses derived from males was significantly higher than that derived from women, yet in both cases losses showed a decreasing tendency over the study period. The lost productivity measured in males in 2017 was of €899 million, while in females it was €284 million.

**Table 2:** Productivity losses (in millions) of lung cancer (sensitivity models 0%; 6%) and percentage of mortality costs of all diseases

Year	Premature mortality costs (baseline)		Premature mortality costs (0%)		Premature mortality costs (6%)	
	*Males*	*Females*	*Males*	*Females*	*Males*	*Females*
2008	1245	319	1278	327	1214	310
2009	1182	244	1213	251	1153	237
2010	1159	228	1190	234	1130	222
2011	1140	219	1170	225	1112	213
2012	1158	174	1189	178	1129	169
2013	1078	170	1107	174	1052	164
2014	1019	164	1045	169	993	161
2015	998	225	1024	231	974	218
2016	963	254	988	261	939	247
2017	899	284	923	291	877	277
Total	10,841	2281	11,126	2342	10,573	2218

**Fig. 2:**
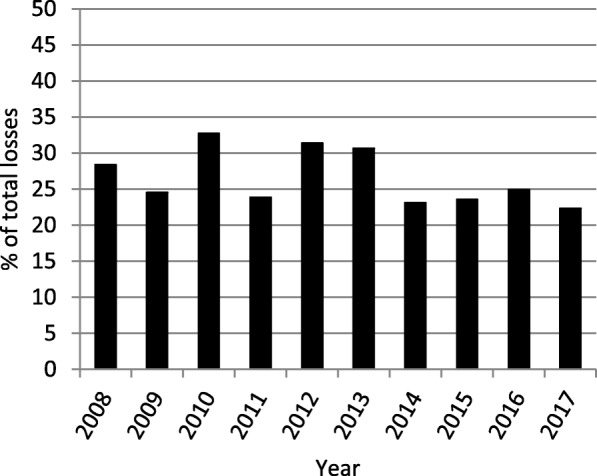
Portion of total cancer-related losses attributable to lung cancer (2008–2017)

Productivity losses derived from total cancer-related mortality were calculated in order to assess this data in context. Lung cancer accounted for 22.33% of the losses in 2017, and lost productivity derived from lung cancer peaked the year 2010 when it represented 32.72% of losses (Fig. 2).

## Discussion

Cancer mortality has been pointed out as one of the major causes of productivity loss [12]. Hence, the increasing interest on measuring and quantifying its economic and social impact.

In general calculations, the productivity loss linked to tumours has been estimated to rise up to hundreds of billions of dollars. The year 2000, the productivity losses associated with cancer only in the United States summed $116 billion and were projected to increase to $148 billion in 2020 [6]. Conversely, in Australia, estimations lower this sum to around $4.2 billion, and 88,000 YPPLLs, presumably an effect of the smaller population [11]. The same study remarked lung cancer as one of the malignancies accounting for the highest portion of costs, in line with former findings in health loss and premature mortality [2]. Lung cancer is, in many countries, the most prevalent cancer type among males, and the principal cancer-related cause of death [17–19]. A study developed in 30 European countries, cancer was appointed responsible for losses of €75 billion, with lung cancer representing 23% of total costs [18], while another study centred in the EU measured €126 billion in losses, with lung cancer representing 15% of the total [19]. In the present study, losses attributable to lung cancer represented 22.33% of all cancer-related losses in 2017.

Similarly, a revision of data in Spain linked cancer to 43.5% of deaths at working age, accounting for 298,753 YPPLL in 2009 and €2.5 billion in losses [10]. Herein, lung cancer alone represented 36,246 YPPLL for males and 23,035 for females in 2017; and its costs summed €1.4 billion that year, €13.1 billion over a ten year period. The differences observed in the number of YPPLL between males and females, as well as the opposed tendencies are presumably derived from the difference in smoking habits and other trends that promote an increasing incidence among women [20]. However, in the calculation of losses, the significantly different wages for males and females in Spain play an important role and distort any interpretation of mortality trends [16].

Interestingly, €2.1 billion in productivity losses were estimated for women with breast cancer in Spain in 2014 [21], an elevated figure presumably due to the prevalence of this cancer type in females. It must be considered that the human capital approach allows an estimation of losses that provides a general vision of disease burden, but comparisons among different studies can become a limitation. In addition, it attributes a greater weight to deaths affecting young working males, versus groups with lower employment rates or income, and thus leading to underestimates and overestimates [16]; its use is nonetheless generalised in the recent literature given its efficacy [22].

On the other hand, further considerations including a calculation of DALYs could be of interest to estimate the social impact of lung cancer, since early onsets of the disease may lead primarily to an incapacity for work.

It is interesting to highlight the increasing incidence of lung cancer-related deaths in females during the study period, a tendency that has been observed at a global scale [23] and continues to pose a challenge for researchers. Nonetheless, a global decreasing tendency of cancer-related deaths has been described, yet important variations are observed per cancer typology [24]. Such trends must be taken into consideration for an adjusted distribution of resources. Equally, the increased years of potential productive life that are lost by the premature death of workers with ages between 50 and 59 years are noteworthy in order to develop adjusted programs.

## Conclusions

The assessment of total productivity losses due to lung cancer provides new information that may assist decision makers in the allocation of resources. Lung cancer supposes a significant burden in Spain, with an increasing tendency in women that is reflected in the increasing number of YPPLL. The promotion of programs aiming to reduce the incidence of lung cancer in working-age individuals will presumably yield substantial reductions in productivity loss counts.

## Data Availability

The data that support the findings of this study are available from the Spanish national statistics institute at http://www.ine.es.

## Abbreviations

DALYs: Disability-adjusted life-years
INE: Spanish national statistics institute (Instituto Nacional de Estadística)
YPPLL: Years of potential productive life lost

## References

[1] Sociedad Española de Oncología Médica. Las cifras del cáncer en España. https://seom.org/dmcancer/wp-content/uploads/2019/Informe-SEOM-cifras-cancer-2019.pdf. Accessed 1 Apr 2019.

[2] Fitzmaurice C, Dicker D, Pain A, Hamavid H, Moradi-Lakeh M, Global Burden of Disease Cancer Collaboration (2015). The Global Burden of Cancer 2013. JAMA Oncol.

[3] Francisci S, Minicozzi P, Pierannunzio D, Ardanaz E, Eberle A, Grimsrud TK (2015). Survival patterns in lung and pleural cancer in Europe 1999-2007: results from the EUROCARE-5 study. Eur J Cancer.

[4] Cancer Research UK. Lung cancer mortality statistics. https://www.cancerresearchuk.org/. Accessed 1 Apr 2019.

[5] Kozielski J, Kaczmarczyk G, Porębska I, Szmygin-Milanowska K, Gołecki M (2012). Lung cancer in patients under the age of 40 years. Contemp Oncol (Pozn).

[6] Oortwijn W, Nelissen E, Adamini S, van den Heuvel S, Geuskens G, Burdof L (2011). Social determinants state of the art reviews - Health of people of working age - Full Report.

[7] Zhang W, Bansback N, Anis AH (2011). Measuring and valuing productivity loss due to poor health: a critical review. Soc Sci Med.

[8] Kigozi J, Jowett S, Lewis M, Barton P, Coast J (2016). Estimating productivity costs using the friction cost approach in practice: a systematic review. Eur J Health Econ.

[9] Yabroff KR, Bradley CJ, Mariotto AB, Brown ML, Feuer EJ (2008). Estimates and projections of value of life lost from cancer deaths in the United States. JNCI J Natl Cancer Inst.

[10] Bradley CJ, Yabroff KR, Dahman B, Feuer EJ, Mariotto A, Brown ML (2008). Productivity costs of cancer mortality in the United States: 2000–2020. J Natl Cancer Inst.

[11] Carter HE, Schofield DJ, Shrestha R (2016). The productivity costs of premature mortality due to cancer in Australia: evidence from a microsimulation model. PLoS One.

[12] Oliva-Moreno J (2012). Loss of labour productivity caused by disease and health problems: what is the magnitude of its effect on Spain's economy?. Eur J Health Econ.

[13] Peña-Longobardo LM, Aranda-Reneo I, Oliva-Moreno J, Vall-Castello J (2015). Pérdidas laborales ocasionadas por muertes prematuras en España: un análisis para el periodo 2005-2009. Rev Esp Salud Pública.

[14] Instituto Nacional de Estadística (INE). Estadística de defunciones según la causa de muerte. http://www.ine.es/dyngs/INEbase/es/operacion.htm?c=Estadistica_C&cid=1254736176780&menu=resultados&idp=1254735573175. Accessed 1 Apr 2019.

[15] Instituto Nacional de Estadística (INE). Encuesta de estructura salarial. http://www.ine.es/dyngs/INEbase/es/operacion.htm?c=Estadistica_C&cid=1254736176918&menu=resultados&idp=1254735976595. Accessed 1 Apr 2019.

[16] Instituto Nacional de Estadística (INE). Encuesta de población activa. http://www.ine.es/dyngs/INEbase/es/categoria.htm?c=Estadistica_P&cid=1254735976594. Accessed 1 Apr 2019.

[17] Pearce A, Bradley C, Hanly P, O’Neil C, Thomas AA, Molcho M (2016). Projecting productivity losses for cancer-related mortality 2011-2030. BMC Cancer.

[18] Hanly P, Soerjomataram I, Sharp L (2015). Measuring the societal burden of cancer: the cost of lost productivity due to premature cancer-related mortality in Europe. Int J Cancer.

[19] Luengo-Fernandez R, Leal J, Gray A, Sullivan R (2013). Economic burden of cancer across the European Union: a population-based cost analysis. Lancet Oncol.

[20] Garrido P, Viñolas N, Isla D, Provencio M, Majem M, Artal A (2019). Lung cancer in Spanish women: the WORLD07 project. Eur J Cancer Care (Engl).

[21] Oliva-Moreno J, Peña-Longobardo LM (2018). Labour productivity loss caused by premature deaths associated with breast cancer: results from Spain over a 10-year period. Breast Cancer Res Treat.

[22] Hanly P, Pearce A, Sharp L (2014). The cost of premature cancer-related mortality: a review and assessment of the evidence. Expert Rev Pharmacoecon Outcomes Res.

[23] Egleston BL, Meireles SI, Flieder DB, Clapper ML (2009). Population-based trends in lung Cancer incidence in women. Semin Oncol.

[24] Cabanes A, Vidal E, Aragonés N, Pérez-Gómez B, Pollán M, Lope V (2010). Cancer mortality trends in Spain: 1980–2007. Ann Oncol.

